# Design, Calibration and Morphological Characterization of a Flexible Sensor with Adjustable Chemical Sensitivity and Possible Applications to Sports Medicine

**DOI:** 10.3390/s24196182

**Published:** 2024-09-24

**Authors:** Alessandro Zompanti, Francesco Basoli, Giovanni Saggio, Francesco Mattioli, Anna Sabatini, Simone Grasso, Martina Marino, Umile Giuseppe Longo, Marcella Trombetta, Marco Santonico

**Affiliations:** 1 Department of Engineering, Università Campus Bio-Medico di Roma, Via Álvaro del Portillo 21, 00128 Rome, Italy; a.zompanti@unicampus.it (A.Z.); a.sabatini@unicampus.it (A.S.); 2 Department of Science and Technology for Sustainable Development and One Health, Università Campus Bio-Medico di Roma, Via Álvaro del Portillo 21, 00128 Rome, Italy; f.basoli@unicampus.it (F.B.); s.grasso@unicampus.it (S.G.); m.trombetta@unicampus.it (M.T.); m.santonico@unicampus.it (M.S.); 3 Department of Electronic Engineering, University of Rome Tor Vergata, Via del Politecnico, 00133 Rome, Italy; saggio@uniroma2.it; 4 Institute for Photonics and Nanotechnologies, National Research Council, Via del Fosso del Cavaliere, 100, 00133 Rome, Italy; 5 Fondazione Policlinico Universitario Campus Bio-Medico, Via Alvaro del Portillo, 200, 00128 Roma, Italy; martinamarino.dr@gmail.com; 6 Research Unit of Orthopaedic and Trauma Surgery, Department of Medicine and Surgery, Università Campus Bio-Medico di Roma, Via Alvaro del Portillo, 21, 00128 Roma, Italy

**Keywords:** wearable sensors, sweat sensors, breath sensors, athlete monitoring, air quality

## Abstract

Active life monitoring via chemosensitive sensors could hold promise for enhancing athlete monitoring, training optimization, and performance in athletes. The present work investigates a resistive flex sensor (RFS) in the guise of a chemical sensor. Its carbon ‘texture’ has shown to be sensitive to CO_2_, O_2_, and RH changes; moreover, different bending conditions can modulate its sensitivity and selectivity for these gases and vapors. A three-step feasibility study is presented including: design and fabrication of the electronic read-out and control; calibration of the sensors to CO_2_, O_2_ and RH; and a morphological study of the material when interacting with the gas and vapor molecules. The 0.1 mm^−1^ curvature performs best among the tested configurations. It shows a linear response curve for each gas, the ranges of concentrations are adequate, and the sensitivity is good for all gases. The curvature can be modulated during data acquisition to tailor the sensitivity and selectivity for a specific gas. In particular, good results have been obtained with a curvature of 0.1 mm^−1^. For O_2_ in the range of 20–70%, the sensor has a sensitivity of 0.7 mV/%. For CO_2_ in the range of 4–80%, the sensitivity is 3.7 mV/%, and for RH the sensitivity is 33 mV/%. Additionally, a working principle, based on observation via scanning electron microscopy, has been proposed to explain the chemical sensing potential of this sensor. Bending seems to enlarge the cracks present in the RFS coverage; this change accounts for the altered selectivity depending on the sensor’s curvature. Further studies are needed to confirm result’s reliability and the correctness of the interpretation.

## 1. Introduction

Several wearable devices and systems are currently commercially available and the research for novel materials, improved sensing and transducing principles of operation, and new applications is rapidly growing [1,2]. Many factors have contributed to the evolution of these devices, including the Internet of Things (IoT) [3], smart materials [4], sensor miniaturization and flexible electronics [5], and low power and energy harvesting [6]. In particular, a smart material can be tailored to sense specific physical or chemical concentrations [7,8]. Additionally, very small and high performance sensors can be designed and fabricated to read and transduce such concentrations through a low-power electronic interface printed on flexible materials [9,10]. These sensors can subsequently be organized into a network, allowing for data transmission to a cloud for real-time analysis and feedback [11,12]. 

Currently, one of the challenges in this field is finding ways to develop an optimal system for each target application. A way to develop such system may be through a bottom-up process going from the modification and adjustment of the chemical sensing mechanism to sensor design and calibration and finally to the development of a large sensor network [13,14]. Generally, applications for health monitoring have been favored by wearables more than other devices [5,15,16]. Examples include real-time disease and treatment follow-up [17], surveillance of fragile individuals [17], and for sport activity monitoring [18,19]. A subset of such devices are chemical sensing wearables. These allow for contemporary monitoring of the subject (via physiological parameters) and the environment by measuring gas and volatile concentrations, temperature, relative humidity (RH), and exhaled breath composition, among other things [20,21]. 

Two published studies [22,23] have previously explored the use of wearable sensors for athlete movement monitoring [22], and traumatic brain injury assessment [23]. The former evaluated the performance of a self-powered, water-resistant, bending sensor using the transverse piezoelectric effect of polypropylene ferroelectret polymer for bending assessments [22]. The latter study reported on a flexible and wearable smart headband based on a polyvinylidene fluoride sensor to evaluate the impact force and acceleration for traumatic brain injury assessment [23]. In both cases it was revealed that via measurements of specific velocity and acceleration parameters, relevant data could be extracted. The headband sensor was proven to be able to provide real-time monitoring of human head impacts, while the wearable sensor, via the extraction of specific velocity parameters, could extract information on bending curvature and speed, which could be applied to athlete monitoring via the creation of movement profiles and comparisons to ideal movement behaviors [22,23]. However, specific bending sensors could go beyond movement and impact force monitoring, as is the case for the sensor currently being studied, which could work toward chemical sensing. 

Chemosensitive wearable sensors, capable of real-time monitoring of oxygen (O_2_) levels and RH, could represent a cutting-edge advancement in sports medicine. These sensors could offer a non-invasive and continuous method of tracking physiological parameters crucial for athlete performance and health [24]. When applied to measuring O_2_ levels, these sensors may provide insights into respiratory efficiency and metabolic demands during physical exertion, aiding in optimizing training regimens and preventing overexertion [24]. Furthermore, monitoring RH, both in the environment and as sweat sensors, can enable the assessment of environmental conditions and facilitate adjustments in hydration strategies to mitigate the risk of heat and dehydration related illnesses and optimize athletic performance [25,26].

Additionally, these sensors may also be applied and designed for breath monitoring, for example those designed for detecting exhaled acetone levels [27]. Acetone serves as a potential biomarker for assessing energy utilization and metabolic efficiency during exercise [28]. By incorporating breath monitors into athlete monitoring protocols, sports medicine professionals could gain unprecedented insights into athletes’ metabolic states in real time [27]. Such information may be used toward the optimization of training regimens tailored to individual metabolic profiles, facilitating targeted interventions to enhance fat utilization and endurance performance [27]. The development and eventual integration of breath monitors in sports medicine has the potential to improve athlete management by enabling personalized interventions informed by real-time metabolic data, thereby maximizing performance potential, and promoting overall health and well-being.

Overall, the goal of a double approach (health–environment) is to correlate the evolution of the physiological and possibly pathological condition of the individual with respect to air quality and changing metabolic parameters [29]. This kind of data fusion is often named exposome [29]. Challenges remain in this field, particularly regarding the low gas concentrations that sensors can detect. Nevertheless, some solutions have been proposed [29]. Overall, the integration of chemosensitive wearable sensors holds promise in the field of sports medicine for enhancing athlete monitoring, training optimization, and overall performance improvement.

The present study investigates a resistive flex sensor (RFS), traditionally used in the sleeves of sensory gloves for measuring finger flexion, applied instead to chemical sensing. This sensor is made of “isles” of carbon particles arranged on a thin plastic strip that, when separating and nearing with flexion and straightening respectively, increase and reduce the values of electrical resistance accordingly. Its arrangement on thin and flexible plastic strips, addressed to favor mechanical bending, make it adaptable for wearable applications. This carbon ‘texture’ has shown to be sensitive to carbon dioxide (CO_2_), O_2_, and RH changes. Moreover, different bending conditions can modulate its sensitivity to these gases and to water vapor. 

Preliminary data on the proposed sensor has already been published [30]. The adjustable sensitivity is a practical response to the variable performance of the prototype sensor depending on the different bending condition and on the target gas. This feature can be taken advantage of as a strategy for correlating health parameters and environmental parameters. For example, the CO_2_ detection limit of the sensor is near the most dangerous threshold indicated for humans but is also in the range useful for exhaled breath monitoring. Thus, a sensor as such would open the way to a further design for exposome evaluation. In the present article, a three-step feasibility study is presented: the design and fabrication of the electronic read-out and control on flexible support, calibration of the sensors to CO_2_, O_2_ and RH, and a morphological study of the material when interacting with the gas and vapor molecules.

## 2. Materials and Methods

### 2.1. Sensing Material

The sensing material of the flex sensors, manufactured by Flexpoint Inc. (South Draper, UT, USA), is composed of isles of carbon particles engineered on a flexible plastic support. RFSs are normally used as a ‘covering’ for fingers or human body parts to measure the flexion and warping of joints or segments of an individual or robot respectively [31].

The RFS tested here is characterized by the following parameters: 0.005 × 0.3 × 3 inches (thickness × width × length), bare-type, carbon-based (isles particles) sensing element.

**Figure 1:**
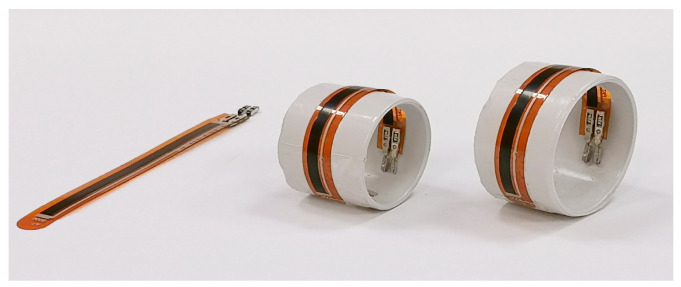
Bending set-up of the RFS; from left to right: flat; 20 mm; 35 mm. The picture also clearly shows Flexpoint’s proprietary carbon/polymer based ink printed on a thin plastic film.

The hypothesis being tested is that the selected RFS could serve as gas sensor for CO_2_, O_2_, and RH, and that its selectivity, sensitivity, and resolution can be modulated by different bending set-ups. Three bending set-ups have been used: flat, 20 mm, and 35 mm of curvature diameter (see Figure 1). 

### 2.2. Read-Out Electronics

**Figure 2:**
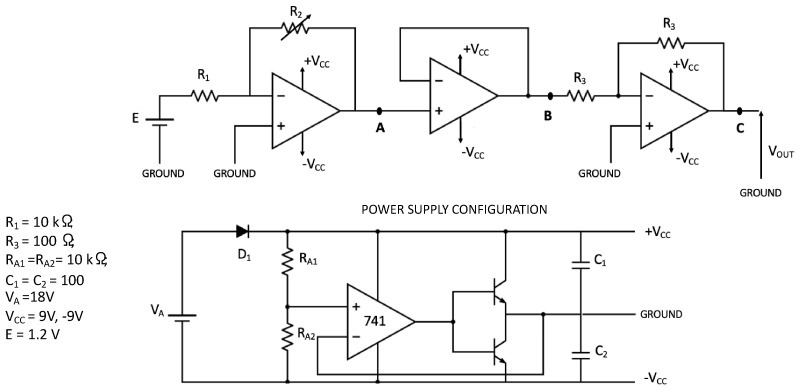
Read-out electronics for the detection of RFS variation. The RFS is the R_2_ in the feedback path of an inverting configuration realized with an opamp TL082 by Texas Instruments. The 3-stage transduction system is completed by a buffer and an inverting stage with unitary gain. The power supply configuration is also reported.

The RFS has been placed into the feedback path of an opamp (TL082 by Texas Instruments, Dallas, TX, USA) in an inverting configuration (Figure 2). E = 1.2 V; Vcc = +/−8.4 V; R_1_ = 10 KΩ; R_2_ is the sensor. The successive two amplification stages (buffer and inverting) are addressed to preserve sensor performance via impedance adaptation and noise reduction (as discussed in the result section).

The resistance values of R_2_ depending on the bending condition are reported in Table 1.

### 2.3. Calibration Set-Up

**Figure 3:**
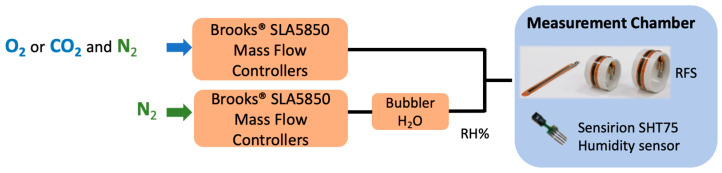
Calibration set-up: CO_2_ and O_2_ are fluxed via the mass flow controllers into the measurement chamber, where the RFS and the humidity sensor are placed.

The calibration set-up is reported in Figure 3. The RFS lodging was thermalized at 25 °C, and the temperature and RH were monitored by a Sensirion SHT75 (Sensirion AG, Stäfa, Switzerland).

CO_2_ and O_2_ were fluxed via the mass flow controllers into the measurement chamber with different concentrations in volume percentage with respect to the nitrogen carrier gas to perform the calibration. 

### 2.4. Prototype Fabrication

**Figure 4:**
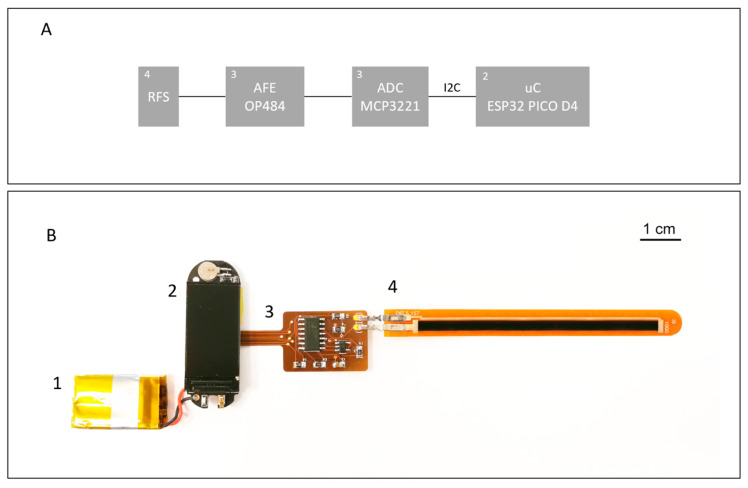
(**A**). Analog front-end of the prototype device coupled with the analog to digital conversion stage and the uC. The voltage output signal coming from the electronic interface is acquired using a 12-bit ADC (MCP3221, MICROCHIP Technology) connected to a uC (ESP32 PICO D4, Espressif Systems) on an I2C bus; (**B**). The flexible sensing system is made of: (1) Li-Po battery; (2) ESP32 uC with Bluetooth/Wi-Fi module; (3) electronic interface on flexible PCB; (4) flexible sensor.

The electronics and the final system represented by the whole measuring chain are reported in Figure 4. The main characteristics of the prototype are reported in Table 2 and Table 3. 

The flexible sensor presented in Section 2.1 is controlled by an ESP32 uC with a Bluetooth/Wi-Fi module, and the relative single supply electronic interface is fabricated on flexible printed circuit board (PCB) and powered by a Li-Po battery.

## 3. Results

### 3.1. Calibration

The results obtained by the calibration tests of O_2_, CO_2_, and RH are presented below. 

#### 3.1.1. Oxygen

Three measurements were executed for each of the following concentration levels of O_2_: 20%, 30%, 50%, and 70%, as described in the methods section, for each of the three bending conditions selected: 0 mm^−1^, 0.05 mm^−1^, and 0.1 mm^−1^. The responses obtained for the first two configurations (0 mm^−1^ and 0.05 mm^−1^) were not significant: they were very low in magnitude and, in general, not sensitive to concentration changes, meaning that the modification of the output voltage of the sensors did not follow the concentration shift.

**Figure 5:**
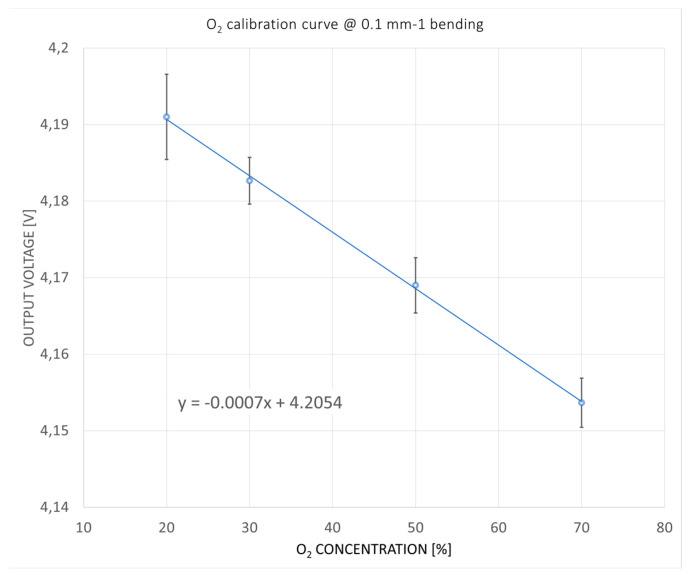
Calibration curve of the O_2_ sensor in the configuration 0.1 mm^−1^. The equation that linearly fits the point dispersion is V= −0.0007 C + 4.2054, where V is the output voltage of the sensor and C is the concentration of the gas. Fit quality is R^2^ = 0.95.

The configuration relative to 0.1 mm^−1^ bending gave very promising results. The curve fitting (shown in Figure 5) is linear, with a good sensitivity of 0.7 mV/%.

#### 3.1.2. Carbon Dioxide

Three measurements were used for each of the following concentration levels of CO_2_: 4%, 10%, 30%, 60%, and 80%, as described in the methods section, for each of the three bending conditions selected: 0 mm^−1^, 0.05 mm^−1^, and 0.1 mm^−1^. As in the case of O_2_, the responses obtained for the first two configurations (0 mm^−1^ and 0.05 mm^−1^) are not significant: they were very low in magnitude and, in general, not sensitive to concentration changes, meaning that the modification of the output voltage of the sensors did not follow the concentration shift.

**Figure 6:**
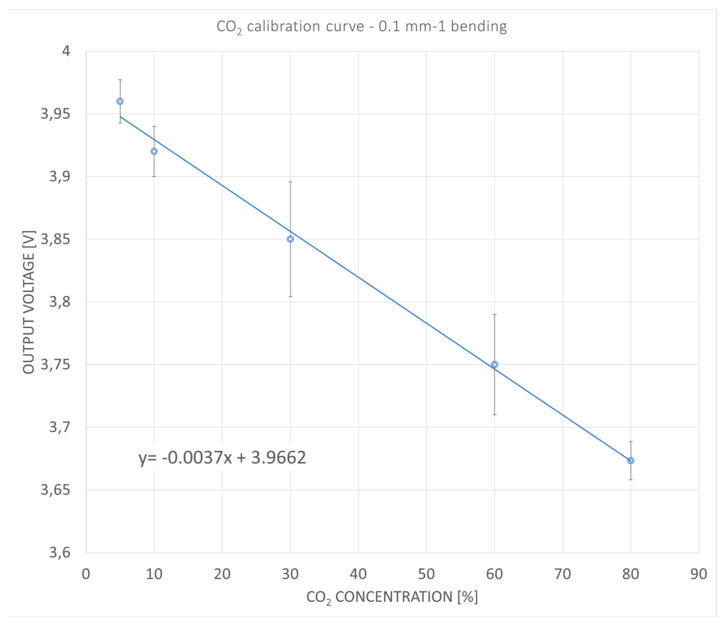
Calibration curve of the CO_2_ sensor, in the configuration 0.1 mm^−1^. The equation that linearly fits the point dispersion is V= −0.0037 C + 3.9662, where V is the output voltage of the sensor and C is the concentration of the gas. Fit quality is R^2^ = 0.94.

The configuration relative to 0.1 mm^−1^ bending gave very promising results. The curve fitting, as shown in Figure 6, is linear, with a good sensitivity of 3.7 mV/%.

#### 3.1.3. Carbon Dioxide and Oxygen

It is important to point out that the sensor was found to respond differently to the bending condition of 0.1 mm^−1^ for O_2_ and CO_2_. The sensor has a unique range of responses to CO_2_ (3.95–3.67 V) and O_2_ (4.19–4.11 V). From these responses, it is possible to establish a specificity in sensing for the two gases. From the sensor’s response, it is possible to predict both the gas and the concentration of the specific gas. In this case, ambiguity is not possible. The slope of each fitting displays the sensor’s sensitivity to a particular gas. In the case of a mixture of gases, we can consider the latter as a third gas that will have different behavior from its components. 

#### 3.1.4. Relative Humidity

The calibration results for RH were presented in a previous preliminary work [27], in which authors highlighted the finding that the RFS in the flat condition shows a non-linear behavior and a saturation trend at RH% > 80%. However, a linear behavior was shown in the overall RH% range in both bending conditions.

The sensitivity (dV/dRH%) depends on the physical deformation of the RFS. In the flat condition, a linear approximation at a low RH% value (less than 10%) accounts for a sensitivity of about 50 mV/%, which is −35 mV/% and −33 mV/% for the 0.05 mm^−1^ and 0.1 mm^−1^ bending, respectively, but within the overall RH% range.

Thus, the RFS shows higher selectivity. However, in the range of 1–40 RH%, the bending configurations provide a lower sensitivity, which is linear within the overall RH% range. These results were confirmed by the calibration performed with the improved electronics read-out circuit presented in the previous section.

### 3.2. Considerations on the Electronic Noise of the Sensor Chain

It is important to note that this kind of sensor is based on changing conductivity as physical transduction, which is affected by noise. This condition means that the effect of electronic noise on the circuit output must be taken into consideration (see Figure 2). 

Considering a range of resistor between 15 Kohm and 310 Kohm, we can estimate the relative Johnson noise:

The spectral density is given by:

(1) $$<v^{2}>={4K}_{B}TR$$

where K_B_ represents the Boltzmann constant while T and R are the temperature (K) and the resistance value (Ω).

For a specific bandwidth:

(2) $$<v_{n}^{2}>={4K}_{B}TR\Delta f\;\mathrm{therefore}:\;v_{n}=\sqrt{{4K}_{B}TR\Delta f}$$

In this case, considering a temperature of 300 K and a bandwidth of 100 Hz:

R_2_ = 15 W; $v_{noise(R_{2})}=4.98\;nV$; R_2_ = 310 K W; $v_{noise(R_{2})}=71\;uV$
and the R_1_ resistor has a voltage thermal noise: $v_{noise(R_{1})}=12.8\;uV$

At this point, in the circuit shown in Figure 2 we can consider R_2_ as being composed of a series of a noise voltage and a resistor voltage. Because the resistor is placed in the feedback path, we can consider the noise voltage directly applied to the output of the first opamp. The resistor R_1_ assumes a constant value of 10 KΩ.

Thus, the first stage will be affected by the following noise voltage:

(3) $$v_{noise\;out\left(A\right)}=\sqrt{v_{noise\_output\left(A;R_{1}\right)}^{2}+v_{noise\_output\left(A;R_{2}\right)}^{2}}$$

where $v_{noise\_output\left(A;R_{1}\right)}^{2}$ and $v_{noise\_output\left(A;R_{2}\right)}^{2}$ are the outputs at point A (Figure 2) when the noise of R_1_ and R_2_ are independently considered. For the calculus, the superposition principle is applied.

(4) $$v_{noise\;out\left(A\right)}=\sqrt{v_{noise\left(R_{1}\right)}^{2}*({\frac{R_{2}}{R_{1}})}^{2}+v_{noise\left(R_{2}\right)}^{2}}$$

The overall value of thermal noise applied to the first stage is:

(5) $$v_{noise\;out\left(A\right)}=72\;uV\;\mathrm{for}\;R_{2}=310\;\mathrm{KOhm}\;\mathrm{and}\;v_{noise\;out\left(A\right)}=19.01\;uV\;\mathrm{for}\;R_{2}=15\;\mathrm{Ohm}$$

The same considerations can be used for the third stage of the circuit:

(6) $$v_{noise\;out\left(C\right)}=\sqrt{v_{noise\_output\left(C;R_{3}\right)}^{2}+v_{noise\_output\left(C;R_{3}\right)}^{2}}$$

which:

(7) $$v_{noise\;out\left(C\right)}=\sqrt{v_{noise\left(R_{3}\right)}^{2}*({\frac{R_{3}}{R_{3}})}^{2}+v_{noise\left(R_{3}\right)}^{2}}=18.09\;nV$$

The low noise of the third stage says that the first stage is more relevant to the calculus of the overall noise. Thus, the resolution of the system can be calculated using (4).

In particular, when the CO_2_ sensitivity is 3.7 mV/%, we can consider a resolution of:

Res_CO2_ = 72 uV/3.7 mV/% = 0.019% for R_2_
The sensitivity to O_2_ is 0.7 mV/% with a resolution of:
Res_O2_ = 72 uV/0.7 mV/% = 0.10%

### 3.3. Sensor Performance

The sensitivities are reported in Table 4: 

For O_2_, the sensitivity is equal to 0.7 mV/%, while for CO_2_, it is equal to 3.7 mV/%. The sensitivity is calculated considering the derivative of the sensor’s response to gas concentration. The response curve, shown in Figure 5 and Figure 6, is obtained as a linear fitting in all working ranges, so the sensitivity is constant for all ranges.

The authors have calculated the RSD% on the range of the sensor’s response to a specific gas at each concentration: for O_2_, the RSD% is equal to 0.13 at 20%, 0.07 at 30%, 0.09 at 50%, 0.08 at 70%, and 0.13 at 80%; for CO_2_, the RSD% is equal to 0.44 at 5%, 0.51 at 10%, 1.19 at 30%, 1.07 at 60%, and 0.42 at 80%.

Moreover, to calculate the LOD, we consider the measurand value for which the S/N ratio is one. Considering a noise of 72 µV and considering the different sensitivities to each gas, the LOD for O_2_ is equal to 0.10%, while for CO_2_ the LOD is equal to 0.019%.

These values are the best resolutions for the sensor, and they are not measurable. The minimum measurable values are generally three times the theoretical value, so the real LOD for O_2_ is equal to 0.30% while for CO_2_ the real LOD is equal to 0.057%.

Several works found in the literature were taken into consideration to evaluate and compare the performances of the flexible sensors studied in this work. The data are reported in Table 5.

### 3.4. Morphological Characterization

The morphological characterization of the sample’s surface was conducted via scanning electron microscopy (SEM) on a new sensor, as supplied by the producer, which was observed both before and after the application of bending stress. In this way it was possible to verify whether, upon the application of stress, there would be morphological or structural changes on the sample’s surface and of the superficial material that could explain the behavior of the sensor.

The apparatus used for this characterization was a Zeiss (Oberkochen, Germany) Evo MA 10 scanning electron microscope equipped with a LaB_6_ thermionic emitter. It allowed us to work with an acceleration voltage in the range 0.2–30 kV, reaching a resolution of 2 nm at 30 kV.

**Figure 7:**
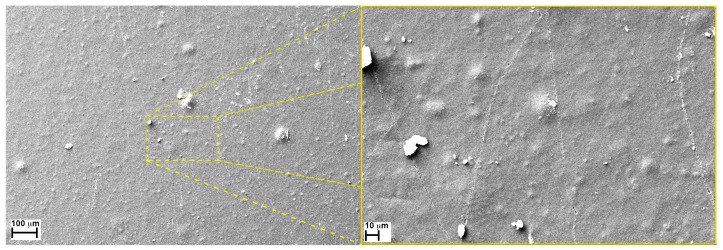
Surface of a new sensor at low (**left**) and high enlargements (**right**), 200 X and 1 KX.

The first images, seen in Figure 7, display the surface of a new sensor at low and high magnification. Even though it is new, it is crossed by a high number of microcracks that, although organized randomly, seems to possess quite a repetitive pattern, at least considering their morphology. This suggests that the presence of these fissures is probably due to the fabrication method of the sensor; a hypothesis confirmed by the sensor’s technology mechanical application design guide [Ref to “the Bend Sensor^®^ Technology Mechanical Application Design Guide” https://flexpoint.com/wp-content/uploads/2021/08/5ec652_ae5e028eb753496eb0608553a7deed1d, accessed on 4 September 2023] which confirms that these microcracks are introduced during processing into the carbon/polymer coating of the sensor. The presence of these microcracks results in their opening and closing upon bending, which enables a change in resistance with deflection and repeatability over the bending process range. In addition to that, the presence of these microcracks allows these sensors to possess better sensitivity in those applications where the presence of a specific environment (presence of high humidity, O_2_, nitrogen, or other gases) might change the device’s response. Which, effectively, most likely happens thanks to the interaction between the said environment and the cracks’ surfaces, where gases can find a larger surface area over which to deposit and react.

To verify this hypothesis and understand how exactly the enhanced behavior of the sensor in the same set of environmental conditions was occurring, superficial characterization was performed on the exact same spot of each sample upon the application of different bending stresses. To do so, holders with different curvatures were 3D printed to apply different and controllable bending stresses on the sensor, especially on the external layer, replicating the working conditions. Three different holder designs were used: a flat one on the new sensor, a holder with a 50 mm diameter as a first bending stress point, and a 20 mm diameter holder to apply a higher flexural stress.

**Figure 8:**
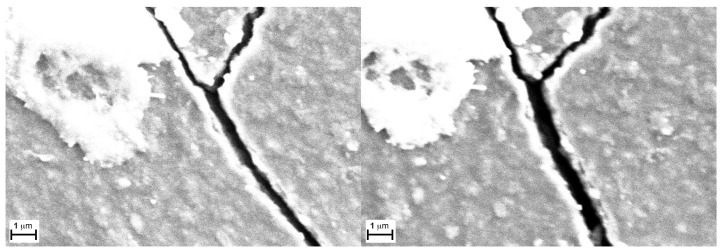
Comparison between a detail of a new sample (**left**) and the sample with the higher bend stress (**right)** applied at two different magnifications (5 KX and 20 KX respectively).

As demonstrated by the images in Figure 8, which compare the new sample to the one with the higher bend stress, even though the new sample’s surface is already cracked, the fissures enlarge upon the application of stress, and the distance between the two edges of the same crack almost doubles in size. 

**Figure 9:**
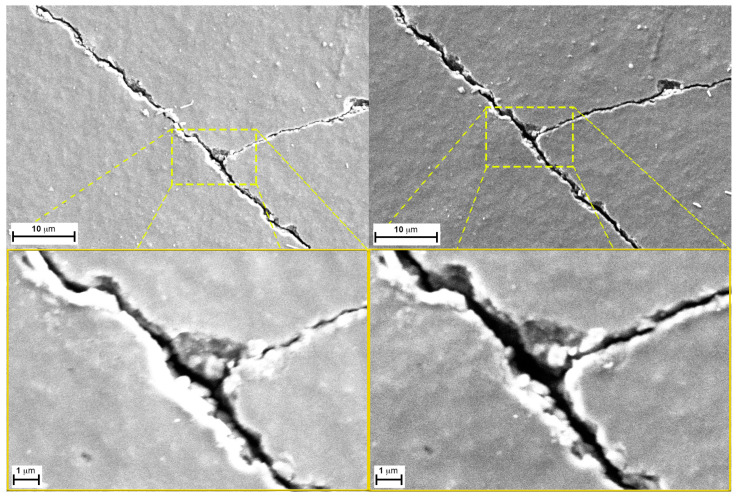
Samples bent over a 50 mm^−1^ (**left**) and 20 mm^−1^ (**right**) holders (magnification 20 KX).

The same behavior repeats in Figure 9, which shows samples bent over the 20 mm and 50 mm holders, respectively. From these findings, we can also infer that the separating gap’s dimensions between the edges is proportional and directly controlled by the applied bending stress.

This could explain the difference in the responses of the sensor when bent. In fact, while the system in the normal unloaded state possesses cracks that are extended in depth and not very separated on the edge, when bent, the cracks’ edges separate but the deeper layers, for geometrical reasons, get closer. Additionally, in the bent material, the cracks’ edges separate further, allowing a higher concentration of environmental gases to enter within the newly formed gaps. This consequentially changes the conductivity of the entire layer, especially considering the high number of cracks present on the surface.

## 4. Discussion

The objective of the present study was to perform an investigation on a resistive sensor, suitable for wearable application and used for the detection of bending conditions, as a chemical sensor exploiting curvature as a parameter to adjust O_2_, CO_2_, and RH sensitivity and selectivity. The results, in light of the achievement of the objectives, must be compared with the alleged benefits provided by the proposed device. As such, the advantages given by this configuration can be summarized by the following points: obtaining a chemical sensor from a physical sensor; obtaining a chemical sensor which is able to monitor gases and vapors for environmental monitoring; managing its sensitivity to diverse analytes in different concentration ranges by operating on the banding configuration; and exploiting the relatively small size, weight, and low power of the RFS to develop a wearable device. Nevertheless, in order to better manage such advantages, a thorough understanding of the sensing mechanism is necessary. 

Results have shown that the RFSs manufactured by Flexpoint Inc. (South Draper, UT, USA) can be used as chemical sensors. The resistance value of the RFS has shown to change in response to modifications of the concentration levels of O_2_, CO_2_, and RH. Table 4 summarizes the results obtained for the three tested bending configurations for each target gas, with reference to the three main parameters: response behavior (linear, not linear), sensitivity, and measured (concentration) range. This table could serve as a map to control the sensor’s functionality and to address further studies. Looking at the characteristics reported in Table 4, it is evident that 0.1 mm^−1^ is the best performing curvature: it is represented by a linear response curve for each of the three gases, the three ranges of concentrations are large enough, and the sensitivity is good for each gas. The other two curvatures are not sensitive to CO_2_ and O_2_ variations, though sensitivity to RH was observed for all conditions. This characteristic could allow modulation of the curvature to be applied to better discriminate RH with respect to the other two gases. In this case, the bending configuration tailors both sensitivity and selectivity.

An area for improvement to be explored in further studies relates to the gas concentrations detected by the device. For example, for the concentration range of CO_2_, the lowest value used in calibration was 4%, which is higher than the maximum concentration of CO_2_ acceptable for an indoor environment. Thus, we encourage the development of devices that can sense a broader range of gas concentrations. A modification of this kind would open the possibility of application to wearable sensors that would enable the simultaneous measurement of CO_2_ in the environment and in exhaled breath, moving in the direction of environment–health applications. Moreover, in the section devoted to electronic noise considerations, the potential of the sensor chain in detecting concentrations down to 200 ppm and 1000 ppm for CO_2_ and O_2,_ respectively, was demonstrated. However, these limits have only been theoretically calculated, hence further calibration measurements will be executed in future experiments.

When considering RFSs for such applications, it is imperative that the bending condition is controlled to tailor sensor functioning. Furthermore, reliable control is also important for understanding the sensor’s mechanism of chemical detection. Based on the present observations made using the captured SEM pictures, it seems that bending enlarges the cracks intrinsically present in the RFS’s coating. Such an interpretation is simple, and it accounts for the sensor’s selectivity depending on the curvature. It is important to note that this selectivity is not very specific given that it is related to the molecule size. Hence, further studies are needed to confirm this interpretation.

Finally, the size and bending variation of this sensor can contribute to its application as a wearable sensor. In future developments, bracelets could be levels. For each bending condition, a specific measurement designed to automatically act on different curvatures to fulfill different monitoring tasks for different gases and concentration could be performed, according to a map, similar to that reported in Table 4, which could be uploaded into a microcontroller to act as an automatic control. 

Tangible applications of this technology include the development of wearable sweat, breath, and environmental sensors applied to the real-time measurement of gaseous compositions, which could provide important physiological and environmental data [25,26]. In sports medicine, gas sensors such as the presented RFS could be programmed to detect and quantify specific respiratory gases such as oxygen and carbon dioxide, which are key indicators of an athlete’s metabolic and respiratory status. Through tailored signal processing algorithms, the sensors could analyze fluctuations in gas concentrations during physical exertion, providing insights into oxygen consumption, carbon dioxide production, and overall respiratory efficiency. This real-time data, when combined with environmental parameters such as temperature, humidity, and air quality, could be used to monitor an athlete’s performance, detect early signs of fatigue, dehydration, or respiratory stress, and optimize training regimes [27,28]. As such, the integration of chemosensitive wearable sensors has the potential to improve the medical management of athletes, thanks to insights into their real-time physical conditions, ushering in an era of personalized and precision sports medicine [27,28]. Before these applications are possible, however, it is worth noting that the concentration ranges of these gases and vapors must be known in order to develop and test corresponding bending configurations able to measure those specific ranges. Such applications would be similar to the previously mentioned studies, which explored the use of wearable sensors for movement monitoring and traumatic brain injury assessment [22,23]. In both of those studies, it was revealed that via measurements of specific velocity and acceleration parameters, relevant data could be extracted. A similar approach would be used for the present RFS sensor, but it would entail gas concentration measurements [22,23]. 

Limitations of the present study include the fact that our experimentation focused on a limited set of gases and concentrations. In the present work, the sensor was calibrated specifically for O_2_ and CO_2_ sensing, with the intent of demonstrating that a specific configuration can be used to detect different gases. Our results confirm that a specific configuration in terms of radius curvature permits the modification of the sensor’s sensibility and specificity for each gas. Additionally, they demonstrate, in this case, that it is indeed possible to obtain the best configuration for a specific mixture (in this case O_2_ and CO_2_) and then obtain the percentage of each gas in a calibrated mixture. With further development of the possible bending configurations, a better understanding of the working mechanism, and further evolution of the RFS’s design, it could be possible to test a variety of different gases. Subsequently, it could be possible to perform sensor training by providing different mixtures of gases in a specific configuration to optimize the S/N ratio. In light of this, a valuable future exploration could focus on the detection of acetone as a metabolic biproduct in breath for application to athlete monitoring. Overall, further experimentation on this device is highly encouraged and could be useful for future application to a variety of health monitoring-related goals. 

## 5. Conclusions

This three-step feasibility study unveiled promising advancements in flexible electronic read-out and control systems for chemical sensing. The optimized RSF curvature of 0.1 mm^−1^ demonstrated superior performance, showcasing linear response curves and good sensitivity across a wide range of concentrations for CO_2_, O_2_, and RH. The proposed working principle, derived from SEM observations, suggests that curvature modulation enhances selectivity by enlarging cracks in the RFS coverage. It is clear that finding out the best curvature tailored for each different gas is possible and that the confounding effect due to RH could be mitigated. Of course, so far, the sensor’s performance with respect to the state of the art is not superior in terms of its parameters, but it has a wide latitude for tailoring sensor performance, thus increasing its usability for specific applications. This means that its performance, at this stage of the research, cannot be evaluated just in terms of ‘numbers’ but must be viewed in terms of its versatility and adjustability. Further studies are necessary to validate these results and confirm the accuracy of the proposed interpretation, driving future advancements in flexible chemical sensing technology. In conclusion, the main result is the confirmation of the tunability of a physical sensor used for chemical detection.

## Figures and Tables

**Table 1:** RFS resistance depending on bending condition.

Bending [mm^−1^]	0	0.05	0.1
Diameter [mm]	0	35	20
Resistance [Kohm]	15	90	310

**Table 2:** Size and material specifications of all of the parts of the prototype: PCB, sensor, uC module, battery.

Sizes and Material Specifications	
Electronics Sizes	
Flex PCB	22 × 16 × 0.1 mm
Flex Sensor	76 × 8 × 0.1 mm
uC Module	38 × 13 × 4 mm
Battery	23 × 14 × 3 mm
Flex PCB Materials	
Substrate	Polyimide (Panasonic Felios Polyimide)
Board Thickness	4 mil (0.1016 mm)
Dielectric	3.2 at 1 MHz
Tensile Modulus	7.1 GPa
Copper Layers	2
Copper Weight	1 oz
Trace Spacing	6 mil (0.1524 mm)
Trace Width	6 mil (0.1524 mm)

**Table 3:** Power specification of the electronic interface.

Power Specification			
uC Power Consumption			
ESP32-PICO-D4			
Mode	Description	Current Consumption	Power Consumption
Active (RF Working)	Transmit BT/BLE	95–100 mA	0.33 W
	Receive BT/BLE		
Modem Sleep	Normal Speed: 80 Mhz	20–31 mA	0.1 W
	(Dual-core chip)		
Sleep Mode	-	0.8 mA	2.7 mW
OP484FSZ			
Spec	Value		
Supply Voltage	3.3 V		
Supply Current	5.4 mA (1.35 × 4)		
Power Consumption	18 mW		
MCP3221A5T			
Spec	Value		
Supply Voltage	3.3 V		
Conversion Current	250 uA		
Standby Current	1 uA		
Active Bus Current	120 uA		
Power Consumption (Worst Case)	0.8 mW		

**Table 4:** Summary of the sensor’s performance in different bending conditions for the three gases tested with reference to linearity, sensitivity, and range.

Bending:		0 mm^−1^	0.05 mm^−1^	0.1 mm^−1^
Target Gas:				
O_2_	Response			Linear
	Sensitivity			0.7 mV/%
	Range			20–70%
RH	Response	Not linear	Linear	Linear
	Sensitivity	50 mV/%	35 mV/%	33 mV/%
	Range	<80%	Full range	Full range
CO_2_	Response			Linear
	Sensitivity			3.7 mV/%
	Range			4–80%
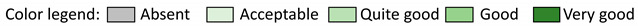				

**Table 5:** Comparison chart of O_2_/CO_2_ resistive sensors.

Reference	Material	Target	Morphology	CO_2_ Concentration (ppm)	Operating Temperature (°C)	Resistance Change (%)
This work	Carbon/Polymer based ink	CO_2_	Thick Film	50,000	25	6
		O_2_		200,000		12
[32]	La_2_O_3_/SnO_2_	CO_2_	Thick Film	1000	400	190
[33]	Sn@CdO	CO_2_	Thick Film	50,000	250	45
[34]	Ag@CuO/BaTiO_3_	CO_2_	Thin Film	5000	300	32
[35]	CuO/CuFe_2_O_4_	CO_2_	Thin Film	5000	250	40
[36]	Ga_2_O_3_	O_2_	Thin Film	200,000	1000	30
[37]	Ce doped Ga_2_O_3_	O_2_	Thin Film	10,000	460	700
[38]	Cerium Oxide	O_2_	Thick Film	400,000	900	13

## Data Availability

The data that supports the findings of the present study is available for retrieval from the corresponding authors upon reasonable request.
